# Hypersensitivity reactions to folinic acid: mechanisms involved based on two case reports and a literature review

**DOI:** 10.1186/s13223-022-00752-5

**Published:** 2022-12-22

**Authors:** Matveï Apraxine, Marc Van den Eynde, Astrid De Cuyper, Françoise Pirson

**Affiliations:** 1 Department of Pulmonology, Cliniques Universitaires Saint-Luc, Brussels, Belgium; 2 Department of Oncology, Cliniques Universitaires Saint-Luc, Institut Roi Albert II, Brussels, Belgium; 3 Centre de l’allergie Saint-Luc, Cliniques Universitaires Saint Luc, Brussels, Belgium; 4 IREC-PNEU, UCL Université Catholique de Louvain, Brussels, Belgium; 5 Department of Pulmonology, GHdC, Charleroi, Belgium

**Keywords:** Folinic acid, Anaphylaxis, Hypersensitivity reaction, Colorectal cancer, Case report

## Abstract

**Background:**

Hypersensitivity reactions (HSR) to antineoplastic agents are an increasing problem, especially when they lead to treatment discontinuation, sometimes without any equivalent therapeutic option. HSR to folinic acid (FA), used particularly for the treatment of digestive carcinoma along with oxaliplatin and 5-fluorouracil, are rare. Only seven publications report HSR to FA, mainly confirmed by the disappearance of symptoms after the withdrawal of FA from chemotherapy. Only two papers describe allergy testing. Due to the difficult diagnosis, patients usually receive several further cycles of chemotherapy with progressively more intense symptoms before the withdrawal of FA.

**Case presentation:**

Here we document two cases of HSR to FA, initially misattributed to oxaliplatin. The first patient described successive cycles with first back muscle pain, then chills and facial oedema and finally diffuse erythema with labial edema despite premedication. The allergy assessment highlighted high acute tryptase levels and intradermal tests positive for FA, pointing to an immunoglobulin E (IgE)-mediated mechanism. The second patient also had lower back muscle pain and chills in addition to tachycardia and desaturation during the administration of FA. Skin tests were negative and tryptase levels normal. After withdrawing FA, the symptoms did not recur, thus allowing the patient to continue chemotherapy. The mechanism of FA hypersensitivity is still unclear. The chronology of symptoms suggests an IgE-mediated mechanism that was not documented in the allergy assessment. A non-IgE-mediated mast cell/basophil activation could be involved, through complement activation or through Mas-related G protein-coupled receptors X2 (MRGPRX2) particularly.

**Conclusions:**

These two cases of anaphylaxis to FA document the clinical manifestations associated with two different mechanisms of HSR. This paper provided the opportunity to review the limited literature on HSR to FA. Through these cases, we hope to draw the practitioner’s attention to FA as a potential agent of severe hypersensitivity, especially if symptoms remain after withdrawing the most suspected chemotherapeutic agents. We want also to stress the importance of allergy testing.

## Background

Hypersensitivity reactions (HSR) [1] to antineoplastic agents are an increasing problem, especially when they lead to treatment discontinuation before the disease becomes refractory to treatment due to the fear of inducing more severe reactions. HSR to carboplatin and oxaliplatin have been reported with incidences ranging from 12 to 17% and more than 50% of patients developing moderate to severe symptoms [2]. Used mainly in colorectal cancer treatment, intravenous folinic acid (FA) is combined with 5-fluorouracil (5-FU) in addition to either oxaliplatin (FOLFOX) or irinotecan (FOLFIRI). FA promotes the binding of fluorodeoxyuridylate, one of the 5-FU metabolites, to thymidylate synthase leading to its inhibition via a covalent ternary complex and enhancing the cytotoxic effect of chemotherapy [3]. HSR to FA are very rare, and only a few cases have been reported in the literature. Due to the higher incidence of HSR to oxaliplatin, FA is not rapidly incriminated. Here we report two cases of immediate hypersensitivity reactions after receiving FA and then review the literature and explore the potential mechanisms involved.

## Case presentation

### Case report 1

A 72-year-old Caucasian male, with no relevant medical history or known drug allergy, was diagnosed with stage IV *KRAS* wild-type colon adenocarcinoma. The patient underwent a left colectomy in May 2019 and initiated first-line treatment with FOLFOX combined with panitumumab (anti-epidermal growth factor receptor monoclonal antibody). After an initial treatment response, maintenance therapy with capecitabine monotherapy was initiated in January 2020. In April 2020, peritoneal metastatic progression was observed. The patient started second-line treatment combining capecitabine, irinotecan, and bevacizumab, but this treatment was rapidly changed to FOLFIRI-bevacizumab due to high-grade diarrhea. In April 2021, following liver metastasis progression, the FOLFOX-panitumumab treatment was rechallenged.

During the fourth cycle, the patient described lower back muscle pain, easily treatable with paracetamol. After the next cycle, at home, he reported shivering, fever, and facial edema. At the sixth cycle, 5 min after beginning the concomitant infusion of oxaliplatin and FA, he developed facial and chest erythema with chills. The infusion was stopped for 2 h. The decision was made to reintroduce chemotherapy on the same day but at a slower speed with premedication consisting of dexchlorpheniramine maleate 5 mg and methylprednisolone 32 mg. Despite premedication, the symptoms reappeared.

During the next cycle, the patient developed immediate diffuse erythema with labial edema and tachycardia. The infusion was stopped. Adrenaline was not required. During this episode, a significant increase in serum tryptase levels was documented, increasing to 16 μg/L from a baseline value of 4 μg/L.

The patient was referred for an allergy consultation. Skin prick tests (SPT) and intradermal tests (IDT) were performed 8 weeks after the first episode and were negative for oxaliplatin, panitumumab, dexamethasone, palonosetron, and pegfilgrastim. The tests were performed at distance of any antihistamine treatment, systemic immunosuppressive drug, local corticosteroid or calcineurin inhibitor.

Practically, for FA skin testing, the commercial form Vorina^®^ 100 mg/4 ml ampoule (also administered during the chemotherapy) is used. The tests begin with SPT performed at 25 mg/mL with a prick lancet on the forearm and associated to the Soluprick negative (glycerinated serum) and positive (histamine dichlorhydrate 10 mg/ml) controls (ALK-Abelló^®^, Almere, Netherlands). The SPT is positive if, after 20 min, the wheal’s largest diameter was  ≥ 3 mm than the negative control. If the SPT is negative, IDT (0.02 mL of serial dilutions) are then performed starting at 25.10^–4^ mg/mL until 2.5 mg/mL with a negative control IDT (NaCl 0.9%). An IDT is positive if after 20 min the wheal’s diameter is  ≥ 3 mm larger than the negative control.

For this patient, SPT for FA was negative but IDT were positive at concentrations of 25.10^–4^ mg/ml and 25.10^–3^ mg/ml. SPT and IDT (until a dilution of 2.5 mg/ml) performed on two control subjects were negative, pointing to a non-irritating reaction for the dilutions used in the patient. A SPT with folic acid 4 mg (Folavit^®^ 4 mg) was also negative. Finally, an oral provocation test (OPT) with folic acid (with successive cumulated doses from 0.12 mg up to 4 mg after 90 min) was negative without an immediate and delayed reaction.

The skin tests and serum tryptase levels documented the mechanism of IgE-mediated hypersensitivity to intravenous FA, without hypersensitivity to natural or synthetic folic acid.

### Case report 2

A 45-year-old female with multiple sclerosis was diagnosed with stage IV *KRAS* mutated caecal adenocarcinoma with ovarian metastases. After a complete resection of the primary tumor and metastases, she started postoperative chemotherapy with FOLFOX. During the 10th cycle, she experienced lower back muscle pain associated with fever and was hospitalized. After recovery, it was decided to stop oxaliplatin, initially incriminated for the side effects, and to keep 5-FU and FA for the remaining treatment cycles. Nevertheless, during the last two cycles, she once again experienced lower back muscle pain and shivering after FA had been running for 10 min. There was no significant increase in serum tryptase levels (8.1 μg/L 30 min after the onset of symptoms with a basal value at 6.9 µg/L).

Six months later, due to metastatic relapse (splenic, pelvic, and lung), the patient had to resume chemotherapy with FOLFIRI-bevacizumab. During the first cycle, while receiving perfusions of both irinotecan and FA, she started to shiver with skin mottling, tachycardia, and cyanosis but without desaturation. The perfusions were stopped, and the patient received intravenous hydration and dexchlorpheniramine. The 5-FU perfusion was subsequently given without difficulties. Serum tryptase levels did not increase after the reaction (7.4 μg/L 1 h after the beginning of the reaction with a basal value at 6.9 µg/L).

SPT and IDR were performed 3 weeks later and were negative for oxaliplatin, cisplatin, carboplatin, bevacizumab, irinotecan, dexamethasone, and FA. The following cycles of chemotherapy omitting FA and 5-FU bolus but containing irinotecan, bevacizumab and continuous infusion of 5-FU were successfully administered, without any suspected reaction.

The negative skin tests, the lack of serum tryptase increase, and the symptoms are suggestive of non-IgE-mediated hypersensitivity.

## Discussion

Two cases of immediate drug hypersensitivity are described here after the intravenous administration of FA. The reactions were initially attributed to oxaliplatin, which was administered concomitantly and has a higher frequency of HSR compared to FA [4]. After the allergy workup, HSR to FA were respectively attributed to IgE-mediated and non-IgE-mediated mechanisms.

Folates are composed of a non-reduced aromatic pteridine ring linked to para-aminobenzoic acid and one or more glutamic residues. Mean dietary intake is around 247–291 μg per day and mainly consists of the polyglutamate form of folic acid, which slowly breaks down into monoglutamates in the small intestine. The synthetic form contains only the monoglutamate form, resulting in higher bioavailability and bypassing the need for fragmentation of the polyglutamate conjugate at the brush border. In the enterocyte, monoglutamate folic acid is reduced, methylated, and released into the bloodstream as 5-methyltetrahydrofolic monoglutamate. However, these mechanisms are saturable, and unmetabolized synthetic folic acid can be detected in blood at doses as low as 200 μg [5]. Folinic acid (5-formyltetrahydrofolate) bypasses the reduction steps required for folic acid, since it is administered intravenously.

The literature describes 27 cases of HSR since 1949, mainly with oral folic acid supplement, ranging from mild anaphylactic reaction to anaphylactic shock. HSR to FA is less often described and mainly reported in patients treated for colon carcinoma. After reviewing previous case reports with HSR to FA (Table 1), we identified only 7 publications describing a total of 12 patients, mainly reported in oncology journals, without any allergy assessment except for the papers by Vermeulen et al. [6] and Ureña-Tavera et al. [7]. For the other five HSR, the involvement of FA was confirmed by the absence of symptoms after withdrawing FA from chemotherapy.

**Table 1:** Case reports of hypersensitivity reactions to folinic acid

References	Age (years), sex	Type of cancer and uneventful treatment	Chemotherapy regimen with folinic acid reaction	Clinical events and number of chemotherapy cycles before FA discontinuation	Diagnosis of HSR to FA
Benchalal et al. [9]	80, M	Stage III colon adenocarcinoma 6 cycles of 5-FU and FA	1st cycle of FOLFIRI	1. Rash 2. Rash, hypotension (R/adrenaline)	No more reactions after FA withdrawal
Vermeulen et al. 1st case [6]	57, M	Metastatic rectal cancer Palliative treatment with 5-FU and FA	1st cycle of 5-FU and FA	1. Urticaria, difficulty breathing	3 months later: SPT + for FA SPT—for folic acid IDT + for FA (200 μg/mL)
Vermeulen et al. 2nd case [6]	59, M	Metastatic colon cancer following a colectomy FOLFOX	1st and 2nd infusion of FOLFOX	1. Urticaria	SPT—for FA IDT + for FA (2 mg/mL) Successful oral desensitization (increased FA doses orally to a dose of 375 mg)
Prabu et al. [20]	16, M	Stage III T-cell lymphoblastic lymphoma MTX and FA	2nd cycle of MTX and FA	1. Chills, rash, fever 2. Rash, vomiting, dizziness, hypotension (R/dopamine)	No argument for sepsis High serum IgE levels of 711 IU/ml (normal: 0.0–158 IU/ml) No more reactions after FA withdrawal
Katirtzoglou et al. [3]	67, F	Stage IV KRAS wild-type colon cancer 18 cycles of FOLFOX6 plus cetuximab	19th cycle of FOLFOX-6 plus cetuximab	1. Facial flushing, dyspnea, cough, hypertension (R/hydrocortisone) 2. Flushing, diarrhea 3. Flushing, dyspnea, hypotension (R/epinephrine, salbutamol)	No more reactions after FA withdrawal (after 3 reactions requiring hydrocortisone or epinephrine)
Damaske et al. [21]	53, M	Stage IV KRAS mutation colon cancer 12 cycles of FOLFOX6 plus bevacizumab 12 cycles of FOLFIRI-bevacizumab 12 cycles of FOLFOX6-bevacizumab	13th cycle of FOLFOX-6 plus bevacizumab	1. Flushing, rash, pruritus 2. Urticaria 3. Headache, facial flushing, generalized pain 4. Pruritus, dyspnea, back pain, generalized discomfort, headache (epinephrine) 5. Hypertension (227/114), facial flushing, headache, severe lower back pain (no epinephrine)	No more reactions after FA withdrawal (after 7 more cycles with discontinuation of 5-FU or oxaliplatin)
Ureña-Tavera et al. 1st case [7]	65, M	Stage IV gastric adenocarcinoma –	1st cycle of FOLFOX	1. Facial erythema, urticaria 2. Urticaria	SPT—for FA IDT—for FA (100 μg/mL) DPT + (urticaria) Normal tryptase levels after DPT
Ureña-Tavera et al. 2nd case [7]	66, F	Stage IV colon adenocarcinoma 17 cycles of FOLFOX	18th cycle of FOLFOX	1. Genital and scalp itching, rhinoconjunctivitis, general malaise 2. Urticaria, rhinoconjunctivitis	SPT—for FA IDT—for FA (100 μg/mL) DPT + (urticaria, rhinoconjunctivitis) Normal tryptase levels after DPT
Ureña-Tavera et al. 3rd case [7]	52, F	Stage IV rectal adenocarcinoma 18 cycles of FOLFOX	19th cycle of FOLFOX	1. Intense chills 2. Chills, back pain, hypertension, fever	SPT—for FA IDT—for FA (100 μg/mL) DPT + (chills, back pain, hypertension, fever) Normal tryptase levels after DPT
Ureña-Tavera et al. 4th case [7]	73, M	Stage IV colon adenocarcinoma -	1st cycle of FOLFIRI	1. Facial erythema, urticaria, eyelid angioedema 2. Urticaria, eyelid angioedema	SPT—for FA IDT—for FA (100 μg/mL) DPT + (urticaria, eyelid angioedema) Normal tryptase level after DPT
Ureña-Tavera et al. 5th case [7]	80, F	Stage IV colon adenocarcinoma 10 cycles of FOLFOX 7 cycles of FOLFIRI	8th cycle of FOLFIRI	1. Dyspnea, chest pain, desaturation, facial erythema 2. Chills, chest pain, facial erythema	SPT—for FA IDT—for FA (100 μg/mL) DPT + (chills, chest pain, facial erythema)
Florit-Sureda et al. [4]	56, M	Stage III sigmoid KRAS wild-type colon cancer 12 cycles of FOLFOX4 FOLFIRI not tolerated 9 cycles of FOLFOX6	10th cycle of FOLFOX6	1. Facial erythema, edema, pruritus, abdominal pain 2. Facial erythema, pruritus, dyspnea 3. Facial erythema, pruritus, dyspnea	No more reactions after FA withdrawal (after 3 more cycles)
This report - case 1	72, M	Stage IV colon KRAS wild-type adenocarcinoma FOLFOX-panitumumab Capecitabine-irinotecan-bevacizumab FOLFIRI-bevacizumab FOLFOX-panitumumab	4th cycle of FOLFOX	1. Lower back muscle pain 2. Chills and facial oedema at home 3. Facial and chest erythema with chills (despite premedication) 4. Diffuse erythema with labial oedema (no adrenaline)	Elevated tryptase levels (16 μg/L; reference value, < 14 μg/L) SPT—for FA IDT + for FA (2.5 μg/mL) SPT and DPT (> 4 mg)—for Folavit
This report - case 2	45, F	Stage IV caecal adenocarcinoma 12 cycles of FOLFOX	> 10th cycle of FOLFOX 1st cycle of FOLFIRI-bevacizumab	1. Lower back muscle pain, chills 2. Lower back muscle pain, chills (during FA administration without oxaliplatin) 3. Chills, cyanosis without desaturation, tachycardia during FA administration	Normal tryptase levels Persistent symptoms despite oxaliplatin discontinuation Normal tryptase levels SPT—for FA IDT—for FA (2.5 mg/mL) No more reactions after FA withdrawal

*5-FU* 5-fluorouracile, *DPT* drug provocation test, *IDT* intradermal test, *IV* intravenous, *FA* folinic acid, *FOLFIRI* FA, 5-FU, irinotecan, *FOLFOX* FA, 5-FU, oxaliplatin, *MTX* methotrexate, *SPT* skin prick test

HSR to carboplatin and oxaliplatin have been reported with high incidences [2]. By contrast, HSR to FA seem to be very rare. However, they could be more frequent than expected, as Ureña-Tavera et al. [7] found a prevalence of 11% (95% confidence interval: 1.98–20.74%) over a 12-month period in their population of FOLFOX- or FOLFIRI-reactive patients. In the absence of allergy assessments, the involvement of FA may be underestimated, especially since the symptoms of HSR to FA can be similar to those with oxaliplatin. As observed in Table 1, patients usually receive several further cycles of chemotherapy (up to five) with progressively more intense symptoms before the successful withdrawal of FA, because FA is rarely suspected. The complexity of regimens with multiple chemotherapeutic agents, sometimes combined with biological agents, makes the diagnosis even more difficult, which highlights the importance of publishing case reports with rare HSR.

Our first case of HSR to FA was due to an IgE-mediated reaction (elevated serum tryptase levels during the reaction and positive IDT), while the second was a non-IgE-mediated reaction. Nevertheless, both clinical situations correspond to anaphylaxis [8]. The mechanism of HSR to FA is still unclear. Serum tryptase levels are currently the best routine biomarker available to assess mast cell activation. Levels are increased with the peak between 1 and 2 h. In 2010, a consensus equation was proposed to diagnose acute mast cell activation: peak tryptase should be  > 1.2 × baseline tryptase + 2 ng/L [8].

It is important to note that HSR to FA can appear either during the first injection (two cases described by Vermeulen et al. [6] and cases 1 and 4 of Ureña et al. [7]) or after several injections (during the 10th administration in our second patient or even after the 18th cycle for case 2 of Ureña et al. [7]).

Different arguments point to an IgE-mediated mechanism. Some patients experienced symptoms such as urticaria, pruritus, hypotension, and tachycardia, suggesting an IgE-mediated anaphylaxis. In this case, tryptase levels can be elevated and skin tests positive. Benchalal et al. [9] even described a case of anaphylactic shock requiring adrenaline. In 2000, Dykewicz et al. [10] showed the existence of IgE antibodies to folate–human serum albumin complex by in-vivo and in-vitro testing. The authors suggested that in IgE-mediated reactions, folic acid, with a molecular weight of only 441 D, probably acts as a hapten by conjugation with self-proteins.

These IgE-mediated reactions are similar to those observed with oxaliplatin, where patients receive several doses before the appearance of the first symptoms, and even more when chemotherapy is interrupted and then resumed again, giving the time for sensitization.

IgE-mediated HSR can also occur on the first exposure to a given drug, as shown for cetuximab in which preexisting IgE antibodies, acquired through tick bites, cause these reactions [11]. It was suggested by Dykewicz et al. [10] that folic acid contained in food could be the sensitizer with clinical cross-reactivity to FA during the first intravenous administration. This hypothesis is ruled out for our first case: the cross-reactivity to folic acid was not confirmed by the SPT and OPT.

Nevertheless, most patients develop non-specific symptoms with lower back pain and chills as well as unelevated serum tryptase levels, and these can sometimes even occur during the first exposure. These reactions are not explained by IgE-mediated HSR.

IgE-independent mechanisms of anaphylaxis include IgG-mediated anaphylaxis, complement activation, direct activation of mast cells by drugs that interact with receptors such as MRGPRX2 and cytokine-mediated mechanisms [12].

Complement activation has been well described with HSR to taxanes. Most patients (80%) reacted after the first or second exposure to taxanes despite the use of standard premedication, with atypical symptoms such as back pain or abdominal pain occurring in around 40% of patients. These reactions are attributed to complement activation by the surfactants used in their formulation (Cremophor EL for paclitaxel and polysorbate 80 for docetaxel) [13]. Cremophor EL promotes the generation of biologically active complement products such as C3a and C5a [14]. These products can activate mast cells resulting in release of histamine, leukotrienes and prostaglandins that can induce flushing, hypoxia, hives and hypotension [15]. However, this mechanism is unlikely since there is no excipient in FA to be incriminated.

Another possible pathway for non-IgE-mediated HSR is the activation of mast cells through MRGPRX2, a new member of the Mas-related G protein-coupled subfamily of receptors, which is present in mast cells and potentially in basophils and eosinophils. MRGPRX2 activation is very effective in activating mast cells, especially with the intravenous administration of drugs at sufficient concentrations to stimulate the receptor and containing structural patterns known as tetrahydroisoquinoline (THIQ). These or similar motifs are found in members of the neuromuscular blocking agents (e.g., cisatracurium) and fluoroquinolone drug family [16]. FA has a relatively similar structure and may activate this receptor, which could explain non-IgE-mediated reactions. Since its first description in 2015 [17], the hypothesis of non-IgE-mediated mast cell activation through MRGPRX2 has been appealing, even if the pathophysiology is not yet completely understood. Nevertheless, it seems that this activation could release more tryptase and less histamine compared to an IgE-mediated activation [18].

IgG-mediated reactions are another possible pathway. IgG-antigen complexes can activate the macrophage and neutrophil low-affinity receptor (FcγRIII) and stimulate platelet-activating factor (PAF). This factor promotes platelet aggregation and release of thromboxane A2 and serotonin, increasing vascular permeability and can lead to hypotension, cardiac dysfunction and smooth muscle contraction. This anaphylaxis has been demonstrated in murines, and it has been hypothesized that in humans IgG antibodies can mediate systemic anaphylaxis if there are large numbers of both IgG and antigen present, which can be the case with parenterally administered drug [19].

Finally, anaphylaxis can be caused by cytokine-release reactions (CRRs), usually triggered by chimeric, humanized or human mAbs and chemotherapeutic agents. The release of proinflammatory mediators such as tumor necrosis factor alpha (TNF-α), IL-1β and IL-6 are responsible for chills, fever and pain, symptoms compatible with the second case-report. It should be mentioned that a mixed reaction can be observed during chemotherapy combining IgE-mediated reactions (redness, pruritus, urticaria, wheezing) and CRRs symptoms (chills, fever, malaise), thereby making it impossible to differentiate between mechanisms [12]. The mixed reaction is probably the most attractive explanation for the first patient.

## Conclusions

Hypersensitivity reactions to FA, commonly used for the treatment of digestive carcinoma along with oxaliplatin and 5-FU, are rare or underdiagnosed, but may lead to discontinuation of chemotherapy. Even if the prevalence of HSR to oxaliplatin is by far the most frequent, it is necessary to be systematic and to test all the molecules administered during the chemotherapy. We documented here two cases of anaphylaxis to FA, one IgE-mediated and the other non-IgE-mediated, thus highlighting the different clinical manifestations.

Through these cases, we hope to draw the practitioner’s attention to FA as a potential agent responsible for HSR, especially if symptoms remain after withdrawing the most suspected chemotherapeutic agents. The diagnostic contribution of the allergy assessment is also demonstrated here.

## Data Availability

All data analyzed for the study are available from the corresponding author on reasonable request.

## Abbreviations

5-FU: 5-Fluorouracil
CRRs: Cytokine-release reactions
FA: Folinic acid
HSR: Hypersensitivity reactions
IDT: Intradermal test
IgE: Immunoglobulin E
MRGPRX2: Mas-related G protein-coupled receptors X2
OPT: Oral provocation test
PAF: Platelet-activating factor
SPT: Skin prick test
